# Anti-α-enolase is a prognostic marker in postoperative lung cancer patients

**DOI:** 10.18632/oncotarget.5316

**Published:** 2015-09-26

**Authors:** Kuan-Chung Hsiao, Neng-Yao Shih, Pei-Yi Chu, Yi-Mei Hung, Jia-Yi Liao, Shao-Wen Chou, Yi-Yuan Yang, Gee-Chen Chang, Ko-Jiunn Liu

**Affiliations:** ^1^ Institute of Clinical Pharmacy and Pharmaceutical Sciences, National Cheng Kung University, Tainan, Taiwan; ^2^ National Institute of Cancer Research, National Health Research Institutes, Tainan, Taiwan; ^3^ School of Medicine, College of Medicine, Fu-Jen Catholic University, New Taipei City, Taiwan; ^4^ Department of Pathology, Show Chwan Memorial Hospital, Changhua City, Taiwan; ^5^ School of Medical Laboratory Science and Biotechnology, Taipei Medical University, Taipei, Taiwan; ^6^ Faculty of Medicine, School of Medicine, National Yang-Ming University, Taipei, Taiwan; ^7^ Division of Chest Medicine, Department of Internal Medicine, Taichung Veterans General Hospital, Taichung, Taiwan; ^8^ Institute of Biomedical Sciences, National Chung-Hsing University, Taichung, Taiwan; ^9^ School of Medicine, China Medical University, Taichung, Taiwan

**Keywords:** lung cancer, ENO1

## Abstract

Our previous studies suggest that antibodies against ENO1 (anti-ENO1 Ab) have a protective role in patients with non-small cell lung carcinoma. In this study, we evaluated the prognostic value of anti-ENO1 Ab levels in non-small cell lung carcinoma patients undergoing surgery. Circulating levels of anti-ENO1 Ab were assessed in 85 non-small cell lung carcinoma patients before and after surgery, and were correlated with clinical outcome. After surgery, patients with a higher increase of anti-ENO1 Ab had a lower hazard ratio and a better progression-free survival. Using animal models, we demonstrated that tumor cells reduce the circulating levels of anti-ENO1 Ab through physical absorption and neutralization of anti-ENO1 Ab with surface-expressed and secreted ENO1, respectively. Mice transplanted with ENO1-overexpressing tumors generated ENO1-specific regulatory T cells to suppress the production of anti-ENO1 Ab. Our results suggest that the increase of anti-ENO1 Ab may reflect anti-tumor immune responses and serve as a prognostic marker in postoperative lung cancer patients.

## INTRODUCTION

Tumor-associated antigens (TAAs) are tumor-expressed antigenic proteins that can trigger immune responses in hosts and can be targeted in clinical diagnosis or cancer therapy [1, 2]. Enolase, a metalloenzyme discovered in 1934 by Lohman and Mayerhof, is involved in the glycolytic pathway and is responsible for the conversion of 2-phosphoglycerate to phosphoenolpyruvate [3]. In addition to its enzymatic function, enolase has been recognized as a biomarker in various diseases [4, 5]. α-enolase (ENO1), one of the major isoforms of enolase in mammals, has been recognized as a multifunctional protein and can be detected in the nucleus and cytoplasm, as well as on the cell surface [6]. Several reports have demonstrated that ENO1 is overexpressed in many types of cancer, including breast, lung, and prostate cancers [7–9]. ENO1 was identified as a TAA in lung and pancreatic cancers [8, 10], and can be detected on the surface of breast, lung, and pancreatic cancer cells [8, 10, 11]. In pancreatic cancer, T cells activated by ENO1-pulsed dendritic cells can lyse pancreatic ductal adenocarcinoma (PDAC) cells *in vitro* and inhibit the growth of transplanted tumor cells in mice [10]. Vaccination of ENO1 in transgenic mice that spontaneously develop PDAC delays tumor progression and enhances survival of mice [12]. In addition, the presence of autoantibodies against ENO1 correlates with longer disease-free survival (DFS) and overall survival (OS) in PDAC patients [13]. These results suggest that immune responses against ENO1 may be beneficial to the host.

We have previously reported that among patients with non-small-cell lung carcinoma (NSCLC), those with tumor cells expressing a higher level of ENO1 have poorer DFS and OS [8]. The plasma level of anti-ENO1 Ab is lower in patients with late-stage NSCLC as compared to that in normal healthy donors and patients with early-stage NSCLC [14]. Recently, we demonstrated that ENO1 on the surface of tumor cells mediates activation of proteolytic enzymes and promotes degradation of the extracellular matrix [15]. Blocking surface-expressed ENO1 with anti-ENO1 Ab, or down-regulation of ENO1 expression by shRNA, significantly suppressed the invasiveness of lung cancer cells *in vitro* and reduced metastasis of lung cancer cells *in vivo* [15]. These results support the notion that immunity against ENO1 may provide anti-tumor effects and result in better clinical outcomes in lung cancer patients.

In this study, we aimed to assess the influence of tumor-associated ENO1 on anti-ENO1 immunity in lung cancer and investigate the relationship between levels of ENO1 Ag in lung cancer cells and levels of anti-ENO1 Ab in the plasma of lung cancer patients. We also evaluated the immunosuppressive activity of tumors on the levels of anti-ENO1 Ab, and the importance of anti-ENO1 Ab on the clinical outcomes of lung cancer patients.

## RESULTS

### The level of anti-ENO1 Ab was increased in most lung cancer patients after surgery

**Figure 1:**
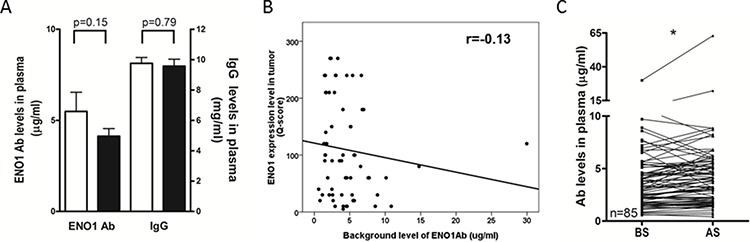
Levels of circulating anti-ENO1 Ab in NSCLC patients before and after tumor removal **A.** Plasma from healthy donors (white bars, *n* = 36) and NSCLC patients (black bars, *n* = 85) were collected and the levels of anti-ENO1 Ab and total IgG were quantified by ELISA. Bars represent mean ± SEM. Statistics were performed using Student's *t* test. **B.** Correlation between the expression of ENO1 in tumors and the level of anti-ENO1 Ab in plasma of patients before surgery. Statistics were performed using Pearson's Correlation Coefficient analysis. **C.** Plasma from the same patients were collected before and 1 month after surgery to remove the tumor. The level of anti-ENO1 Ab in the plasma was determined by ELISA. Each line indicates the change in the plasma level of anti-ENO1 Ab in the same patient (*n* = 85) before and after surgery. Statistics were performed using one tailed paired *t*-test. *P < 0.05. AS, after surgery; BS, before surgery; ENO1, α-enolase.

To investigate the impact of tumor mass on the immune status of lung cancer patients, we examined the level of anti-ENO1 Ab before and after surgery. In Figure 1A, there is no statistically significant difference in the level of total IgG and anti-ENO1 Ab in the plasma of patients before surgery and normal donors. To evaluate the influence of tumor-associated ENO1 on the level of anti-ENO1 Ab, we examined the association between ENO1 expressed in the tumor and anti-ENO1 Ab in the blood from the same patients before surgery. As shown in Figure 1B, there is a negative correlation (*r* = −0.13) between the expression of ENO1 in tumor sections and the blood level of anti-ENO1 Ab. Chi-square tests reveal that the expression of ENO1 in tumors correlates negatively with the level of anti-ENO1 Ab (*P* = 0.025), but not with other clinical variables (Table 1). There is no significant correlation between the level of anti-ENO1 before surgery and other clinical variables (Supplemental Table 1). We further compared the level of anti-ENO1 Ab before and 1 month after surgery in the same patients. As shown in Figure 1C, the level of anti-ENO1 Ab was significantly increased 1 month after surgery in most patients. This result suggests that the existence of a tumor mass influences the level of anti-ENO1 Ab in the blood.

**Table 1:** Correlation of patients' clinical variables and the expression of ENO1 in tumor section (Q score)

Variable	Total^A^, *n* (%)	>100, *n* (%)	≤100, *n* (%)	*P*
**Sex**				
Male	25 (40.3)	9 (36.0)	16 (64.0)	0.568
Female	37 (59.7)	16 (43.2)	21 (56.8)	
**Age (y)**				
≤65	40 (64.5)	17 (42.5)	23 (57.5)	0.637
>65	22 (35.5)	8 (36.4)	14 (63.6)	
**Pathology**				
Adeno	54 (87.1)	21 (38.9)	33 (61.1)	0.799
Non adeno	8 (12.9)	4 (50.0)	4 (50.0)	
**pTNM stage**				
I/II	49 (79.0)	18 (36.7)	31 (63.3)	0.264
III	13 (21.0)	7 (53.8)	6 (46.2)	
**Tumor Size**				
≤5 cm^3^	31 (50.0)	16 (51.6)	15 (48.4)	0.07
>5 cm^3^	31 (50.0)	9 (29.0)	22 (71.0)	
**Anti-ENO1 Ab (BS)**				
≤3.3 μg/ml	29 (46.7)	16 (55.2)	13 (44.8)	0.025
>3.3 μg/ml	33 (53.3)	9 (27.3)	24 (72.7)	
**EGFR mutation**				
Yes	35 (62.5)	14 (40.0)	21 (60.0)	0.577
No	21 (37.5)	10 (47.6)	11 (52.4)	

A Results of 62 out of 85 patients that were followed-up for 2 years. The mean Q-score was 100. The median level of anti-ENO1 Ab before surgery was 3.3 μg/ml. The median tumor volume is 5 cm^3^.

Adeno, adenocarcinoma; Non adeno, non-adenocarcinoma; y,years; BS, before surgery.

**Figure 2:**
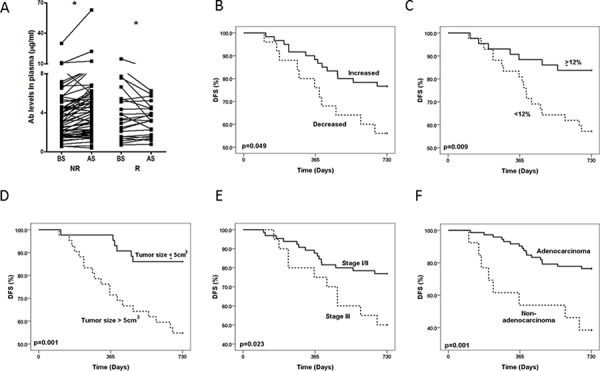
Changes in the level of anti-ENO1 Ab in patients with or without recurrence and Kaplan-Meier analysis of 2-year DFS **A.** Plasma from the same patients were collected before and 1 month after surgery. The level of anti-ENO1 Ab in the plasma was determined by ELISA. Each line indicates the change in the plasma level of anti-ENO1 Ab in the same patient before and after surgery. NR: patients with no tumor recurrence in two years (*n* = 60); R: patients with tumor recurrence in two years (*n* = 25). AS, after surgery; BS, before surgery. Statistics were performed using one tailed paired *t*-test. *P < 0.05. Kaplan-Meier analysis of 2-year DFS was stratified according to: **B.** the variation in the level of anti-ENO1 Ab before and one month after surgery. **C.** Lower (<12%) or higher (≥12%) increase of anti-ENO1 Ab. **D.** Tumor size (≤5 cm^3^ and > 5 cm^3^). **E.** pTNM stage (stage I/II and stage III). **F.** Histology subtype (adenocarcinoma and non-adenocarcinoma).

Since approximately 30% of NSCLC patients who undergo curative-intent surgery develop recurrence, and 50–90% of recurrences occur within the first two years after surgery [16], we next analyzed the correlation between the incidence of cancer recurrence and the changes in the level of anti-ENO1 Ab before and 1 month after surgery in patients with 2 years of follow ups. As shown in Figure 2A, a significant increase in the level of anti-ENO1 Ab was observed in patients without cancer recurrence, while the anti-ENO1 Ab level was reduced in those with recurrence. Kaplan-Meier survival analysis indicates that patients with an increased level of anti-ENO1 Ab after surgery have a better 2-year DFS than those with a decreased Ab level (Log Rank test; *P* = 0.049) (Figure 2B).

We further used the median value (12% increase) of the variation of the anti-ENO1 Ab level of all patients before and after surgery as a cutoff value to evaluate the correlation of the increase of anti-ENO1 Ab level with the clinical variables and disease outcome in these patients. As shown in Table 2, there is no statistical difference in patients with a higher (≥12%) or lower (<12%) increase of anti-ENO1 Ab after surgery with regards to gender, histological subtypes, pTNM stages, tumor volumes, or EGFR mutation status. There is only a correlation with the patient age; of the patients is correlated, however, the reason for this correlation is currently unknown.

**Table 2:** Correlation of patients' clinical variables and increase of anti-ENO1 Ab in plasma after surgery

Variable	Total, *n* (%)	≥12%, *n* (%)	<12%, *n* (%)	*P*
**Sex**				
Male	38 (44.7)	20 (52.6)	18 (47.4)	0.735
Female	47 (55.3)	23(48.9)	24 (51.1)	
**Age (y)**				
≤65	56 (65.9)	23 (41.1)	33 (58.9)	0.015
>65	29 (34.1)	20 (69.0)	9 (31.0)	
**Pathology**				
Adeno	72 (85.9)	36 (50.0)	36 (50.0)	0.799
Non adeno	13 (14.1)	7 (53.8)	6 (46.2)	
**pTNM stage**				
I/II	65 (76.5)	36 (55.4)	29 (44.6)	0.111
III	20 (23.5)	7 (35.0)	13 (65.0)	
**Tumor Size**				
≤5 cm^3^	43 (50.6)	21 (48.8)	22 (51.2)	0.744
>5 cm^3^	42 (49.4)	22 (52.4)	20 (47.6)	
**EGFR mutation**				
Yes	46 (61.3)	24 (52.2)	22 (47.8)	0.97
No	29 (38.7)	15 (51.7)	14 (48.3)	

The median tumor volume was 5 cm^3^. The median increase of anti-ENO1 Ab was 12%.

Adeno, adenocarcinoma; Non adeno, non-adenocarcinoma; y, years.

Kaplan-Meier survival analysis reveals that NSCLC patients with a higher increase of anti-ENO1 Ab (≥12%), a tumor volume less than the median value (≤5 cm^3^), diagnosis of early stages (stage I/II), and adenocarcinoma subtype of lung cancer, have a significantly better 2-year DFS (Log Rank test; *P* = 0.009, *P* = 0.001, *P* = 0.023 and *P* = 0.001 respectively; Figure 2C–2F). The hazard ratio, which was adjusted in the multivariate Cox regression model for tumor progression, is lower in patients with a higher increase of anti-ENO1 Ab (*P* = 0.011), adenocarcinoma subtype (*P* = 0.017) and a small tumor volume (*P* = 0.017) (Table 3). Similar results were obtained when we used 10%, 15% or 20% of increase in the anti-ENO1 Ab level after surgery as a cutoff value to analyze the prognostic role (DFS) of anti-ENO1 Ab in Cox regression analysis (Supplemental Table 2). In addition, a higher increase of anti-ENO1 Ab (10%, 12%, 15% and 20%) was correlated with better DFS and overall survival (OS) in stage I patients (*n* = 54, Supplemental Table 3 and 4, and Supplemental Figure 1). This correlation was not observed in stage III patients (data not shown). Furthermore, the increase in the level of anti-ENO1 Ab is better as an independent factor in predicting overall survival as compared to other biomarkers in stage I patients (Supplemental Table 4).

**Table 3:** Multivariate Cox regression analysis to adjust the risk factors for tumor progression

Prognostic factor	β	HR	95% CI	*P*
**Histological subtype**				
Non adeno vs Adeno	1.057	2.88	1.205–6.880	0.017
**pTNM stage**				
III vs I/II	0.386	1.47	0.627–3.452	0.375
**Anti-ENO1 level**				
<12% vs ≥12%	1.168	3.22	1.302–7.944	0.011
**Tumor size**				
>5 cm^3^ vs ≤5 cm^3^	1.194	3.30	1.235–8.826	0.017

The median tumor volume was 5 cm^3^. The median increase of anti-ENO1 Ab was 12%.

Adeno, adenocarcinoma; Non adeno, non-adenocarcinoma; β, regression coefficient; CI, confidence interval; HR, hazard ratio.

### The existence of tumors accelerated the clearance of anti-ENO1 Ab

**Figure 3:**
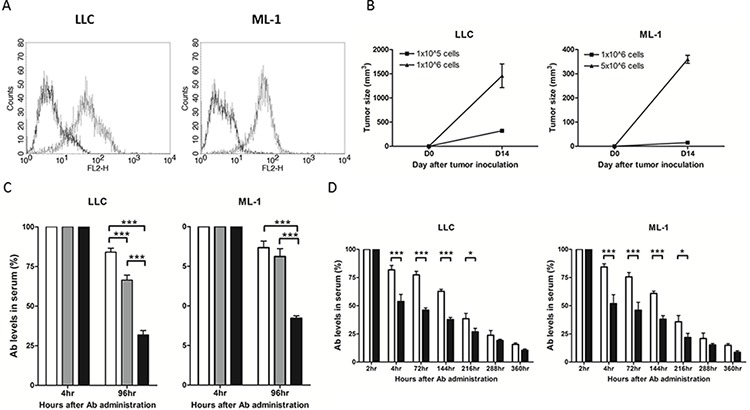
The level of anti-ENO1 Ab in sera of mice challenged with tumor cells or administered with soluble ENO1 **A.** Surface expression of ENO1 in LLC and ML-1 cells. Solid line: isotype control; dotted line: anti-ENO1 Ab. **B.** Mice (*n* = 5) were s.c. challenged with different numbers of tumor cells on day 0 and the tumor size on day 14 was measured. Left panel: B6 mice with LLC inoculation; right panel: BALB/c mice with ML-1 inoculation. Mean ± SEM is shown. **C.** Anti-ENO1 Ab was administered i.v. into tumor-free mice or mice (*n* = 5) with different sizes of tumors on day 14. The level of anti-ENO1 Ab detected 4 h later was set to 100%, and the level of anti-ENO1 Ab 96 h after Ab administration was determined. Left panel: B6 mice with LLC inoculation; right panel: BALB/c mice with ML-1 inoculation. White bars: tumor-free mice; gray bars: mice with small tumor; black bars: mice with large tumors. Mean ± SEM is shown. **D.** Anti-ENO1 Ab was i.v. administered into B6 (left panel) or BALB/c mice (right panel) (*n* = 10) on day 0 followed by i.p. injection of ENO1 2 h later. The levels of anti-ENO1 Ab in the sera collected at different time points were determined. The level of anti-ENO1 Ab 2 h after Ab administration was set to 100%. White bar: mice with PBS injection; black bar: mice with ENO1 injection. Mean ± SEM is shown. *P < 0.05, and ***P < 0.001 by *t*-test (C–D).

We conducted experiments with murine tumor models to elucidate the potential molecular and cellular mechanisms responsible for the modulation of anti-ENO1 immune response in cancer patients in our clinical studies. In our animal experiments, we employed a murine lung cancer cell line, LLC, and a murine hepatoma cell line, ML-1, both expressing substantial amounts of ENO1 on the surface (Figure 3A) [15]. We first investigated the influence of tumor mass on the level of adoptively transferred anti-ENO1 Ab. Different amounts of LLC or ML-1 cells were s.c. injected into the right flank of the C57BL/6 or BALB/c mice on day 0 to allow the formation of tumor masses with different sizes.

Once tumors had become evident on day 14 (Figure 3B), we adoptively transferred 400 μg of monoclonal anti-ENO1 Ab by i.v injection and then measured the Ab clearance rate in the circulation. The detected level of anti-ENO1 Ab in mice with large tumor sizes was significantly less than that in mice with small tumor sizes on day 4 after Ab transfer (Figure 3C) in both tumor models. We observed that a substantial amount of adoptively transferred anti-ENO1 Ab accumulated at the tumor area 24 h after i.v. injection (Supplemental Figure 2) [15], which may be due to absorption of transferred Ab by tumor cells through surface ENO1.

### Administration of soluble ENO1 reduced the detected level of adoptively transferred anti-ENO1 Ab

To determine the possible influence of ENO1 released by tumor cells on the detected level of anti-ENO1 Ab in the circulation, we first estimated the amount of ENO1 released by 1 × 10^6^ tumor cells using a competitive ELISA assay (Supplemental Figure 3). Results from the competitive ELISA suggested that 400 μg of extracellular ENO1 was required to achieve the extent of reduction of anti-ENO1 Ab level mediated by large tumors after 96 h (52~69% reduction of anti-ENO1 Ab in both groups, Figure 3C). Therefore, we i.p. injected either 400 μg of recombinant soluble ENO1 or PBS as a control to naïve C57BL/6 or BALB/c mice 2 h after i.v. injection of 400 μg of anti-ENO1 Ab, and then measured the detectable level of the transferred anti-ENO1 Ab at different time points. The level of anti-ENO1 Ab in the group of mice with recombinant ENO1 injection was lower than that in the control group (T_1/2_ of anti-ENO1 Ab clearance was 181 h in the control group and 102 h in ENO1 administration group, Figure 3D).

### Growth of tumor cells overexpressing ENO1 reduced the production of anti-ENO1 Ab

**Figure 4:**
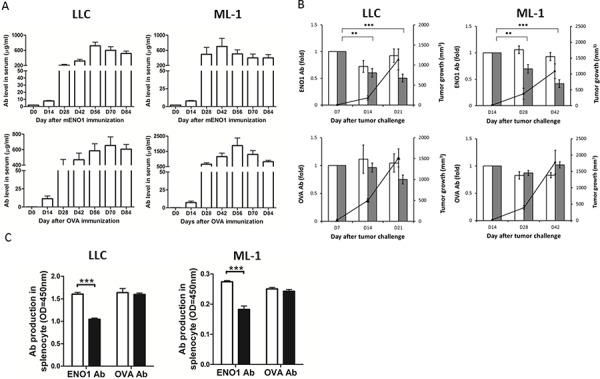
The impact of tumor growth on the Ab response against ENO1 **A.** Mice (*n* = 10) were immunized with ENO1 (top) or OVA (bottom) on day 0, and boosted on days 14 and 21. Sera were colledcted every 14 days and the levels of anti-ENO1 and anti-OVA Ab were determined. Left panels: B6 mice; right panels: BALB/c mice. Mean ± SEM is shown. **B.** When anti-ENO1 (top) and anti-OVA Ab (bottom) levels were steadily maintained, ML-1 (right) or LLC cells (left) were s.c. implanted into mice (*n* = 5). Tumor growth (right Y axis) and the serum level of anti-ENO1 or anti-OVA Ab (left Y axis) were measured periodically. White bar: Ab level in tumor-free group; Gray bar: Ab level in tumor-implanted group; ▲: tumor growth. Mean ± SEM of triplicate experiments is shown. **P < 0.01, and ***P < 0.001 by *t*-test. **C.** Splenocytes from ENO1 or OVA-immunized mice (*n* = 5), with or without tumor challenge, were collected on day 42 after ML-1 implantation or day 21 after LLC implantation and the production of anti-ENO1 or anti-OVA Ab was determined. Left panel: B6 mice with (black bars) or without (white bars) LLC inoculation; Right panel: BALB/c mice with (black bars) or without (white bars) ML-1 inoculation. ENO1, α-enolase; OD, optical density. Mean ± SEM is shown. ***P < 0.001 by *t*-test.

We next immunized naïve mice with either mouse ENO1 or an unrelated Ag, OVA, and determined the level of anti-ENO1 and anti-OVA Ab before and after the implantation of LLC or ML-1 cells. When the amount of anti-ENO1 and anti-OVA Ab in the blood reached a constant level around day 84 (Figure 4A), we implanted s.c. LLC and ML-1 cells into mice vaccinated with either ENO1 or OVA, and followed the changes in the level of anti-ENO1 or anti-OVA Ab, respectively. As shown in Figure 4B, the level of anti-ENO1 Ab in tumor-implanted mice with ENO1-immunization was reduced significantly during the exponential growth period of both tumors, while the level of anti-OVA Ab in tumor-implanted mice with OVA-immunization remained at a similar level.

We further determined that there is a significant reduction in the production of anti-ENO1 Ab by splenocytes of mice vaccinated with ENO1 and transplanted with LLC or ML-1 cells. No reduction of anti-OVA Ab was observed in tumor-bearing mice vaccinated with OVA (Figure 4C).

### Treg cells isolated from ENO1-overexpressing tumor suppressed the proliferation of ENO1-specific CD4^+^ T cells

**Figure 5:**
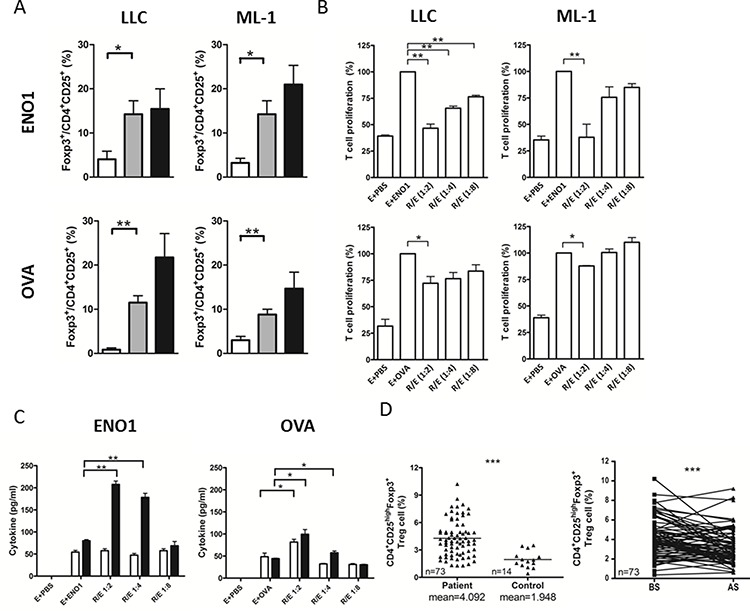
The suppressive ability of tumor-associated Treg cells in splenocyte proliferation assay **A.** Mice (*n* = 5) immunized with ENO1 (top) or OVA (bottom) were challenged with LLC (left) or ML-1 cells (right) as described above. The percentage of CD4^+^CD25^+^Foxp3^+^ Treg cells in the isolated lymphocyte was determined. White bars: lymphocytes from spleens of tumor-free mice; Gray bars: lymphocytes from spleens of tumor-bearing mice; Black bars: lymphocytes from tumor. **B.** B6 (left) or BALB/c mice (right) (*n* = 10) were immunized with ENO1 (top) or OVA (bottom). Splenocytes (as T effector cells) from immunized mice were cultured with ENO1 (E + ENO1) or OVA (E + OVA) with or without Treg cells, isolated from tumor at 1:2, 1:4 and 1:8 (Treg:effector) ratios. The proliferation of CFSE-labeled CD4^+^ T cells was determined. The proliferation in the group of E + Ag was set to 100%. E + PBS: splenocytes only. **C.** Splenocytes from ENO1 or OVA immunized mice were cultured with ENO1 or OVA and tumor-associated Treg cells. The amounts of IL-10 (white bars) and TGF-β (black bars) in the culture supernatant were determined. *P < 0.05, and **P < 0.01 by *t*-test (A–C). Bars represent Mean ± SEM (A–C). **D.** The percentage of CD4^+^CD25^high^Foxp3^+^ Treg cells in NSCLC patients (*n* = 73) and healthy donors (*n* = 14) BS (left) and the changes BS and AS (right) were determined. AS, after surgery; BS, before surgery. The horizontal lines indicate mean. ***P < 0.001 by one tailed paired *t*-test. R: Treg cells, E: splenocytes.

To clarify the role of Treg cells in the reduction of anti-ENO1 Ab, we first evaluated the amount of Treg cells in the tumor-implanted mice. In mice vaccinated with ENO1 or OVA, the percentage of FoxP3^+^ Treg cells in total CD4^+^CD25^+^ cells of spleens from tumor-bearing mice was significantly increased compared to those from tumor-free mice (Figure 5A). There was also a high percentage of FoxP3^+^ Treg cells in total CD4^+^CD25^+^ cells isolated from the tumors.

We examined the specificity of Treg cells isolated from the tumors for their ability to suppress the proliferation of ENO1- and OVA-specific CD4^+^ T cells. As shown in Figure 5B, splenocytes from ENO1- and OVA-immunized mice proliferated vigorously in the presence of ENO1 and OVA, respectively. FoxP3^+^ Treg cells isolated from LLC or ML-1 tumors significantly suppressed the proliferation of ENO1-specific CD4^+^ T cells in a Treg/Effector ratio-dependent manner. These Treg cells suppressed the proliferation of OVA-specific CD4^+^ T cells only at the highest Treg/Effector ratio, and the suppression was much less than that observed with the ENO1-speficific CD4^+^ T cells. No significant suppression on the Ag-dependent proliferation of ENO1- or OVA-specific CD4^+^ T cells was observed using Treg cells isolated from the spleens of tumor-free mice (Supplemental Figure 4).

Treg cells were reported to exhibit suppression activity through cell-cell contact and secretion of immune-modulating cytokines such as TGF-β and IL-10 [17, 18]. Figure 5C demonstrates that the levels of TGF-β are significantly higher in the culture supernatant of Ag-activated splenocytes co-cultured with tumor-associated Treg cells. The levels of TGF-β appear to be higher in the culture containing tumor-associated Treg cells and ENO1-specific CD4^+^ T cells as compared to that containing OVA-specific CD4^+^ T cells. These results indicated that a higher amount of TGF-β was produced by Treg cells during Ag-specific interaction with CD4^+^ T cells.

To verify our observation in animal tumor models, we further analyzed the numbers of CD4^+^CD25^High^Foxp3^+^ Treg cells from another group of NSCLC patients. The number of CD4^+^CD25^High^Foxp3^+^ Treg cells was significantly higher in the blood of patients than that of normal donors before surgery (Figure 5D, left panel). The number of circulating Treg cells was significantly decreased 1 month after surgery in most patients (Figure 5D, right panel). These results confirm the impact of tumor mass on the number of Treg cells in lung cancer patients.

## DISCUSSION

The theory of tumor immunosurveillance was first described by Burnet and Thomas in 1970 [19], following the concept proposed by Ehrlich [20]. They proposed that the host immune system recognizes and eradicates transformed cells to prevent carcinogenesis in the body. However, the immune-editing hypothesis suggested that tumor cells may survive under conditions of tumor immunosurveillance through three steps: elimination, equilibrium, and escape [21]. The presence of a tumor mass indicates that tumor cells escape from immune rejection and survive in the host. Immune escape of cancer cells may involve several different mechanisms, which are contributed by tumor cells, immune cells and stromal cells [22]. Tumor cells can avoid the activation of T cells or prevent the recognition of cytotoxic T lymphocytes (CTLs) by shedding or downregulating the expression of molecules that are involved in antigen processing and presentation [23, 24]. In addition, tumor cells can directly interfere with the immune system by releasing immunosuppressive factors or recruiting immunosuppressive cells, such as CD4^+^CD25^+^ Foxp3^+^ Tregs into the tumor microenvironment [25, 26].

In our previous studies, ENO1 was identified as a TAA in NSCLC patients, and the expression of ENO1 on the surface of cancer cells has been also described [8]. Autoantibodies aroused by TAA can be used for cancer detection, and most of the literature recognizing autoantibodies as tumor markers describes elevated serum levels of autoantibodies in cancer patients [27–29]. However, the serum level of anti-ENO1 Ab in late-stage patients with lung or breast cancer has been found to be lower as compared to those in healthy donors, and the level of anti-ENO1 Ab in advanced stage NSCLC patients has been shown to be lower than that of early-stage patients [14]. It is unknown why late-stage NSCLC patients have a lower level of anti-ENO1 Ab. The existence of immunosuppressive status has been described in NSCLC patients [30], suggesting that the reduction of anti-ENO1 Ab in serum may result from the suppression of anti-ENO1 immune response in late-stage NSCLC patients.

Several possible mechanisms are likely to be responsible for this phenomenon. Firstly, necrosis of tumor cells under hypoxic conditions during tumor growth may release cell-associated proteins such as ENO1. When soluble ENO1 Ag is released into the tumor microenvironment or circulation, it can interact with anti-ENO1 Ab and form an immune complex, which is promptly cleared by macrophages in the liver or spleen [31, 32], resulting in a lower level of circulating anti-ENO1 Ab in cancer patients with tumor cells overexpressing ENO1. Secondly, anti-ENO1 Ab can be absorbed and bound to surface-expressed ENO1 in tumor cells, leading to a reduction of blood anti-ENO1 Ab in cancer patients. ENO1 of *Streptococcus sobrinus* is an immunosuppressive protein that suppresses T-dependent Ag-induced immune responses in mice [33]. The production of IL-10 was observed after i.p injection of recombinant *Streptococcus sobrinus* ENO1 in mice. Since ENO1 is highly conserved from prokaryotes to eukaryotes [34], ENO1 in eukaryotes may share the same immunosuppressive ability as that in prokaryotes.

ENO1 from necrotic cancer cells is released to tumor-infiltrating cells, such as lymphocytes, dendritic cells, and macrophages during tumor growth. In a tumor microenvironment, these cells can create an immunosuppressive condition that promotes tumor progression after interacting with autoantigens [35, 36]. Indeed, in our study, we observed substantial amounts of Treg cells in tumors of mice and the circulation of cancer patients before surgery. We also demonstrated that the FoxP3^+^ Treg cells isolated from ENO1-overexpressing tumors exhibit ENO1-specific immunosuppression. Taken together, our results support the notion that ENO1 secretion may represent an abnormal process evolved by tumors as a mechanism of immune escape (to elude targeting of this TAA).

In this study, we demonstrated that the anti-ENO1 immune response was suppressed during tumor growth, and the tumor volume in ENO1-immunized mice was smaller than that in control mice (Figure 4B). These results suggest that an active anti-ENO1 immune response may provide benefits to the host. Using the level of anti-ENO1 Ab and the number of Treg cells in patients as surrogate indicators, the reversion of immunosuppressive status was observed in most NSCLC patients after curative-intension surgery in this study. Removal of tumor may lead to a reduction in the number of Tregs and circulating ENO1. After surgery, patients with a higher increase of anti-ENO1 Ab had a lower hazard ratio and a better clinical outcome. This is also true for patients with stage I disease. Thus, in this study we demonstrated that the increase of anti-ENO1 Ab after surgery serves as an independent prognostic marker for NSCLC patients. We believe this result has valuable impact beyond the standard clinical prognostic markers and suggests that providing sufficient amount of anti-ENO1 Ab after surgery may provide clinical benefit for patients with early-stage disease.

## MATERIALS AND METHODS

### Patients and plasma

This was a single-center prospective observational study. Patients were recruited for this study under the Institutional Review Board of Taichung Veterans General Hospital. Patients with completely resected stage I to IIIA NSCLC, according to the 7th edition of the American Joint Committee for Cancer staging system, from September 2008 to April 2012 in Taichung Veterans General Hospital, were enrolled [37]. After surgical resection, all patients underwent follow-up every 1 to 3 months with chest radiography or computed tomography scan to assess disease progression until death or end of study period. Lung cancer tumor tissues and plasma specimens were collected for testing. Plasma was collected at baseline before surgery, and at 1 month after surgery. The patient characteristics are listed in Supplemental Table 5. The 36 healthy control samples used in this study were collected as previously described [14]. For the plasma specimens, 3–5 milliliters of whole blood were drawn into vacuum blood collection tubes with EDTA as the anticoagulant. The plasma was centrifuged for 7 min at 2000 rpm, stored at −80°C until further analysis, and the blood cells were subjected to regulatory T (Treg) cell staining. The tumor volume in patients was calculated using a previously described formula: [4/3 × π × (length/2)^3^] [38]. Tumor specimens were obtained for *EGFR* mutation analysis as previously described [39].

### Staining of human treg cells

The Treg cells in peripheral blood mononuclear cells (PBMCs) of patients or healthy donors were stained following the manufacturer's instruction of the Human Regulatory T Cell Whole Blood Staining Kit (eBioscience, San Diego, CA) and analyzed using the FACS Calibur™ flow cytometer (Becton Dickinson, Franklin Lakes, NJ).

### Animals and cell lines

C57BL/6JNarl (B6) and BALB/c mice (males, 5–6 weeks old) were purchased from the National Laboratory Animal Center and maintained in the animal facility of the National Health Research Institutes. The murine hepatoma cell line, ML-1, was maintained in RPMI-1640 with 10% FBS (Invitrogen, Carlsbad, CA) [14]. The murine Lewis lung carcinoma (LLC) cell line was obtained from American Type Culture Collection (ATCC) and cultured in DMEM supplemented with 5% FBS.

### Antibodies

The mouse monoclonal Ab against ENO1 used in animal experiments and surface staining of ENO1 in LLC or ML-1 cells was produced as described previously [14]. The mouse isotype-control Ab was obtained from LTK BioLaboratories (Taoyuan, Taiwan). Allophycocyanin (APC)-conjugated rat anti-mouse CD4 and corresponding isotype control Ab were purchased from BD Biosciences (Becton Dickinson). Rabbit anti-ENO1 Ab and associated isotype control were purchased from Genetex (San Antonio, TX). Biotin-conjugated goat anti-mouse IgM/G/A Ab was purchased from Millipore (Billerica, MA).

### ENO1 Ag preparation

For animal experiments and splenocyte proliferation assay, glutatione S-transferase (GST)-tagged mouse ENO1 was generated and purified as previously described [8]. The mouse ENO1 used in the ENO1 direct-coating ELISA was derived by removing the GST tag of purified GST-tagged ENO1, using a Thrombin CleanCleave™ kit (Sigma-Aldrich, St Louis, MO) as previously described. [8] The human ENO1 used in the ENO1 direct-coating ELISA was purchased from Abcam (San Francisco, CA).

### ELISA

To detect the presence of anti-ENO1 or anti-OVA Ab in the patients' plasma or mouse sera, 96-well plates were directly coated with 50 μl of ENO1 (human or mouse, 6.5 μg/ml in PBS) or OVA (Sigma-Aldrich) (10 μg/ml in PBS) overnight at 4°C. The plates were then washed 3 times with PBS containing 0.05% Tween 20 (PBST) and blocked with 3% BSA (Sigma-Aldrich) in PBS (BSA/PBS) at room temperature for 1 h. After blocking and washing, plasma or sera diluted in 1% BSA/PBS (1:100), a serial diluted anti-ENO1 Ab or anti-OVA Ab (as a standard concentration) were added to the wells for incubation at room temperature for 2 h. After washing, HRP-conjugated goat anti-mouse IgG or HRP-conjugated goat anti-human IgG (Jackson ImmunoResearch, West Grove, PA) diluted in 1% BSA/PBS (1:10,000) was added to all wells at room temperature for 1 h. After enzymatic activity had been initiated by incubation with 3,3′,5,5′-tetramethylbenzidine (TMB) (Thermo Scientific, Rockford, IL) for 15 min at room temperature, the extent of the reaction was quantified by spectrophotometry (Spectra Max^®^ M5, Molecular Devices, Sunnyvale, CA) at an OD of 450 nm. The increase of anti-ENO1 Ab one month after surgery in patients' plasma was calculated as: [(plasma level of anti-ENO1 Ab)_one month after surgery_ − (plasma level of anti-ENO1 Ab)_before surgery_)/(plasma level of anti-ENO1 Ab)_before surgery_] × 100. Human total IgG was determined using a Human IgG ELISA Quantitation Set (Bethyl, Montgomery, TX) according to the manufacturer's instructions.

### Quantification of the expression of ENO1 in tumor sections

Immunohistochemical staining and Quick score methods (Q-score) were used to detect and quantify the expression of ENO1 in tumor sections as previously described [8, 40]. Briefly, the intensity of staining was scored as 0, 1, 2, and 3 for negative, weak, moderate, and strong staining, respectively. The proportion of staining positivity in tumors was scored as 0–100%. The Q-score was calculated to obtain a score ranging from 0 to 300 with the following formula: Q-score = (Score of staining intensity) × (Score of proportion of staining positivity) × 100.

### Quantification of Ab released by splenocytes

The 96-well filtration plates (Multiscreen, Millipore) were coated with 10 μg/ml ENO1 or OVA protein overnight at 4°C and blocked with 3% BSA in PBS (200 μl/well) at room temperature for 2 h. After washing with PBS, splenocytes harvested from mice immunized with mouse ENO1 or OVA (with or without tumor challenge) were seeded into plates (2 × 10^5^ cells/well). After overnight incubation at 37°C, plates were washed with PBS and incubated with biotin-conjugated goat anti-mouse Ig Ab (1:5000) in 1% BSA/PBS at room temperature for 2 h. Plates were washed with PBST and incubated with streptavidin-HRP (Becton Dickinson) (1:1000) in 1% BSA/PBS for 30 min at room temperature. After washing with PBST, plates were incubated with TMB for 15 min, and the extent of the reaction was quantified by spectrophotometry (Spectra Max^®^ M5) at an OD of 450 nm.

### FACS analysis

For surface staining of ENO1 in ML-1 and LLC cells, 1 × 10^6^ cells were washed 3 times with PBS and then stained with 1 μg anti-ENO1 Ab or isotype control at 4°C for 1 h. After washing with PBS, cells were stained with FITC-conjugated goat anti-mouse IgG Ab (1:200) at 4°C for 1 h. Cells were washed once more with PBS, then suspended in 1% paraformaldehyde in PBS and analyzed by flow cytometry. To detect the mouse Treg cells in the spleen, splenocytes from tumor-bearing or tumor-free mice were isolated and stained with a mouse regulatory staining kit (eBioscience) following the manufacturer's instructions.

### The effect of tumor mass on the serum level of anti-ENO1 Ab

In order to study the effect of tumor volume on the serum level of anti-ENO1 Ab, groups of BALB/c mice were s.c. implanted with 5 × 10^6^ ML-1 cells on their the right flanks on day 0 or with 1 × 10^6^ ML-1 cells on day 7; and groups of C57BL/6 mice were s.c. implanted with 1 × 10^5^ or 1 × 10^6^ LLC cells on day 0. The tumor volume was estimated using the modified ellipsoid formula (π/6 × [length × width^2^]) [15]. On day 14, 400 μg of anti-ENO1 Ab was adoptively transferred into groups of tumor-bearing and tumor-free mice by i.v. injection. The sera were collected at each indicated time point, and the levels of anti-ENO1 Ab were determined by ELISA.

### Mice immunization

Groups of BALB/c or C57BL/6 mice were first s.c. immunized with either 20 μg ENO1, 20 μg OVA (Sigma-Aldrich), or PBS emulsified in CFA (Sigma-Aldrich) on day 0; and then boosted twice with the same Ag (10 μg/mouse) or PBS emulsified in Incomplete Freund's Adjuvant (IFA) (Sigma-Aldrich) on day 14 and day 21. Sera were collected from immunized mice every 14 days, and the levels of anti-ENO1 or anti-OVA Ab were determined by ELISA. Once the serum level of anti-ENO1 and anti-OVA Ab had reached a steady state (day 84), 1 × 10^6^ ML-1 cells or 1 × 10^5^ LLC cells were s.c. implanted to the right flanks of BALB/c or C57BL/6 mice, respectively. The tumor growth and serum level of anti-ENO1 or anti-OVA Ab were measured every 7 days for LLC-challenged mice and every 14 days for ML-1-challenged mice, based on the growth rate of these two cell lines *in vivo*.

### Splenocyte isolation

Splenocytes from Ag immunized mice, with or without tumor inoculation, were isolated by pressing the spleen through a 100 μm cell strainer (Becton Dickinson). After treatment with an erythrocyte lysis buffer (eBioscience), the remaining cells were washed with PBS and resuspended in either 1% BSA/PBS for splenocyte proliferation assay or RPMI-1640 containing 10% FBS for Treg cell isolation.

### Mouse treg cell isolation

To determine the percentage of Treg cells in the tumor, single cell suspensions were obtained by treating tumor fragments with collagenase (0.1 mg/ml, Sigma-Aldrich) at 37°C for 20 min and then pressing the cells through a 100 μm cell strainer (Becton Dickinson). The lymphocyte population in the single cell suspensions was harvested by density gradient centrifugation using Ficoll^®^-Paque (GE Healthcare, Piscataway, NJ) with density equal to 1.084 [41]. The harvested lymphocyte population was either stained with a mouse regulatory staining kit to determine the percentage of Treg cells by flow cytometry as described above, or subjected to the isolation of tumor-associated Treg cells using the EasySep™ Mouse CD4^+^CD25^+^ Regulatory T Cell Isolation Kit (STEMCELL, Vancouver, Canada). The Treg cells in the spleens of mice, with or without tumor challenge, were isolated using a similar procedure.

### Splenocyte proliferation assay

Splenocytes, suspended in 1% BSA/PBS (1 × 10^7^ cells/ml), were labeled with CFSE (1 μM in cell suspension) (Life technologies, Grand Island, NY) at 37°C for 10 min. After adding a 5-fold volume of ice-cold RPMI-1640 containing 10% FBS into the cell suspension to terminate CFSE labeling, CFSE-labeled cells were harvested by centrifugation. After washing three times with 5-fold volume of ice-cold RPMI-1640 containing 10% FBS, the CFSE-labeled cells were resuspended in RPMI-1640 containing 10% FBS, 50 μM 2-ME (Sigma-Aldrich) and 1 mM HEPES (Sigma-Aldrich). The splenocytes were then cultured in the U-bottom wells of a 96-well plate (1 × 10^6^ cells/well) with OVA or ENO1 (20 μg/ml) for 7 days or Concanavalin A (Con A) (Sigma-Aldrich) (5 μg/ml) for 3 days as a positive control. After a 7-day incubation, the culture supernatant of each well was collected for future analysis, and the cells were harvested, stained with APC-conjugated anti-mouse CD4 Ab, and analyzed by flow cytometry. The reduction of CFSE intensity in CD4^+^ T cells was used to determine the proliferation of effector cells. For immunosuppressive experiments, the CFSE-labeled cells were cultured with either OVA or ENO1 (20 μg/ml) as the stimulating Ag in the absence or presence of Treg cells, isolated either from the tumors of tumor-bearing mice or the spleens of tumor-free mice, at Treg/splenocytes (effector) ratios of 1:2, 1:4, and 1:8. After a 7-day incubation period, the cells were harvested, and the proliferation of CFSE-labeled CD4+ T cells was determined as described above.

### IL-10 and TGF-β detection

Mouse CBA Flex sets (Becton Dickinson) were used to detect the concentration of IL-10 and TGF-β in the culture supernatants collected from the splenocytes proliferation assay following the manufacturer's protocol.

### Statistics

The comparison of data collected from different groups were analyzed by a Student's *t* test using GraphPad Prism 4.0 software (GraphPad Software, Inc., La Jolla, CA) and presented as mean ± SD values. The variance of ENO1 Ab in the same patients before and after surgery was analyzed by a paired Student's *t* test using Graphpad Prism 4.0 software. The Chi-square test was used to compare the clinical and pathological characteristics of patients with the increase of anti-ENO1 Ab level or the expression of ENO1 in tumor sections (Q-score). Spearman's Rank test, Chi-square test, and the Pearson's Correlation Coefficient were used to evaluate the correlation between the expression level of ENO1 in tumors and the background level of anti-ENO1 Ab in plasma. The 2-year DFS ratio was calculated using Kaplan-Meier analysis. Multivariate Cox regression analysis was used to identify independent predictors of 2-year DFS and OS. All clinical patient data were analyzed using SPSS 17.0 software (IBM Corporation, Armonk, NY). A *P* value of ≤ 0.05 was considered statistically significant. *P < 0.05, **P < 0.01 and ***P < 0.001.

### Study approval

This study was approved by the Institutional Review Board of Taichung Veterans General Hospital. Written informed consent for genetic testing and clinical records was received from participants prior to inclusion in the study. All animal experimental protocols were approved by the Institutional Animal Care and Use Committee of the National Health Research Institutes before the initiation of the study.

## SUPPLEMENTARY DATA, METHODS FIGURES AND TABLES


