# Supplementary material for: Anti-α-enolase is a prognostic marker in postoperative lung cancer patients
Source: Oncotarget. 2015 Sep 26;6(33):35073–86. doi: 10.18632/oncotarget.5316 (PMC4741510; doi:10.18632/oncotarget.5316)
Supplement: Supplementary file 1 [file oncotarget-06-35073-s001.pdf]

SUPPLEMENTARY DATA

SUPPLEMENTARY METHODS

Competitive ELISA

We used competitive ELISA to evaluate the effect of soluble ENO1 Ag on the detection of anti-ENO1 Ab. The wells of 96-well plates were coated with 50 µl of ENO1 (10 µg/ml) overnight at 4°C and blocked with 3% BSA in PBS at room temperature for 1 h. Different concentrations of recombinant ENO1 protein (0.75–48 µg/ml) were mixed with 1 µg anti-ENO1 Ab (to set up a competitive ELISA standard curve) before adding to the wells, and the plates were incubated at room temperature for 1 h. After washing, HRP-conjugated goat anti-mouse IgG diluted in 1% BSA/PBS at a dilution of 1:10,000 was added to all wells, and the plates were incubated at room temperature for 1 h. After enzymatic activity had been initiated by incubation with 3,3′,5,5′-tetramethylbenzidine (TMB) (Thermo Scientific, Rockford, IL) for 15 min at room temperature, the remaining concentration of anti-ENO1 Ab after competition was determined by ENO1 ELISA as described in Methods.

Preparation of fluorescence-labeled Ab

The anti-ENO1 Ab and corresponding isotype Ab were labeled with Alexa Fluor 488 dye (Molecular Probes, Eugene, OR) and purified with size exclusion purification resin as previously reported (15).

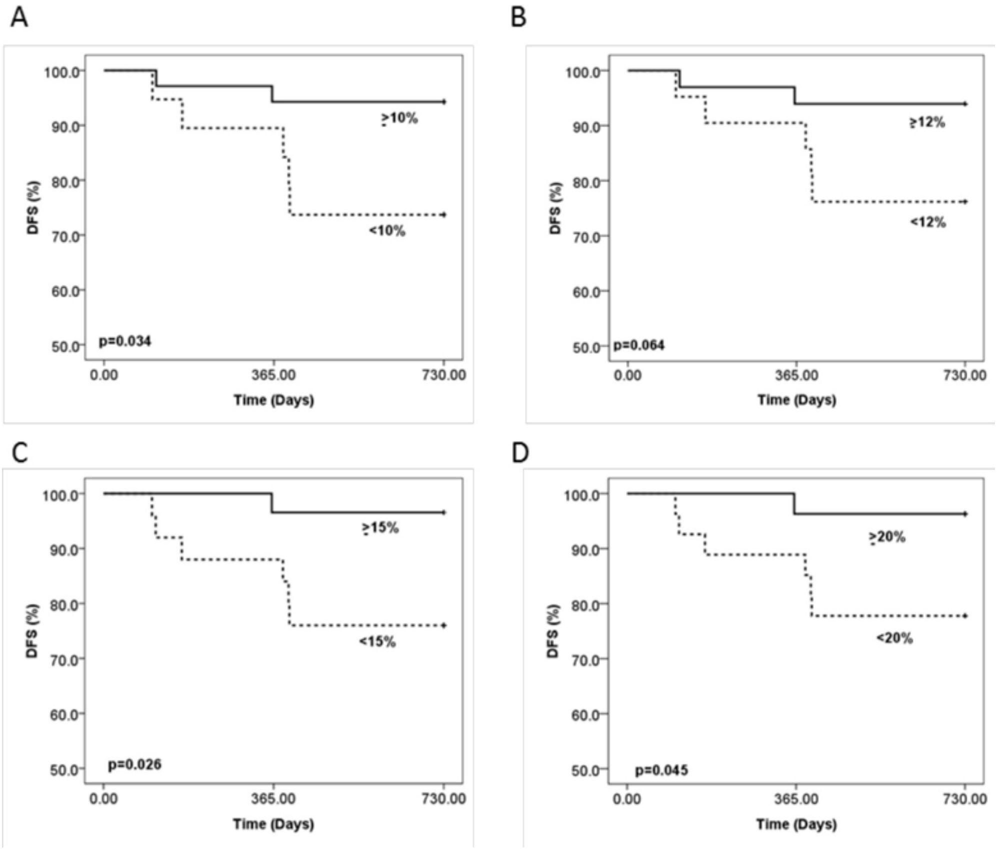

Supplementary Figure S1: Kaplan-Meier analysis of 2-year DFS in patients with stage I disease. Kaplan-Meier analysis of 2-year DFS was stratified according to the variation in the level of anti-ENO1 Ab: **A.** 10% increase of anti-ENO1 Ab. **B.** 12% increase of anti-ENO1 Ab. **C.** 15% increase of anti-ENO1 Ab. **D.** 20% increase of anti-ENO1 Ab.

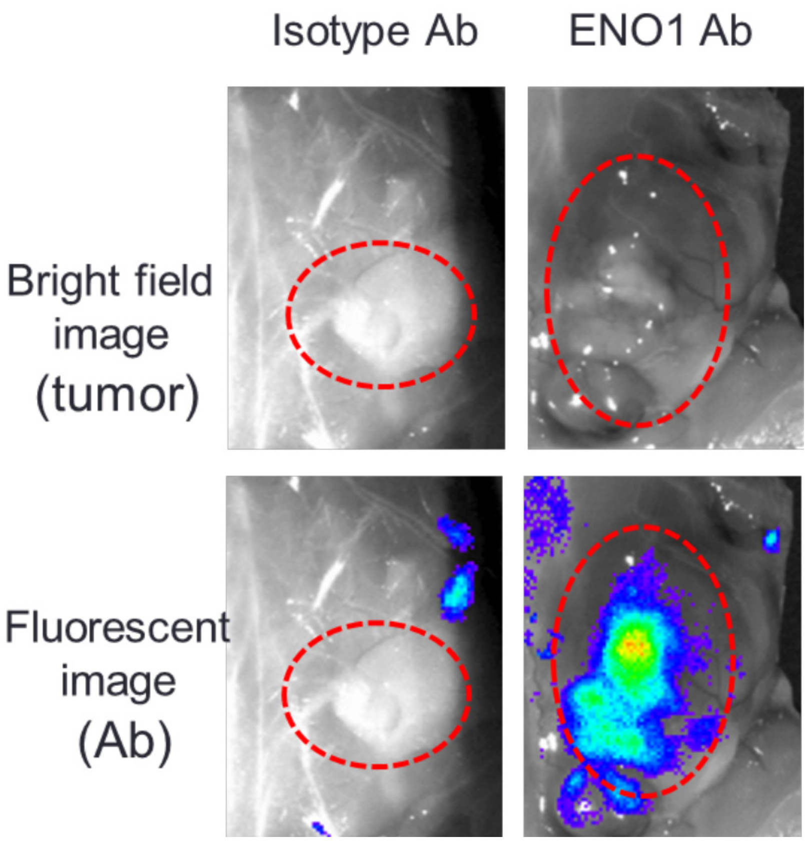

Supplementary Figure S2: Adoptively administered anti-ENO1 Ab was accumulated at tumor site. ML-1 cells (1×10^6 cells/mouse) were s.c. injected into BALB/c mice (*n* = 5). After tumor volume had reached 400 mm^3, 100 µg Alexa Fluor 488-labeled anti-ENO1 Ab (ENO1 Ab) or isotype control Ab (Isotype Ab) was i.v. injected into mice. The fluorescent intensity (indicating the presence of Ab) was detected 24 h later by the IVIS System. The location of the tumor was indicated by the dotted circle.

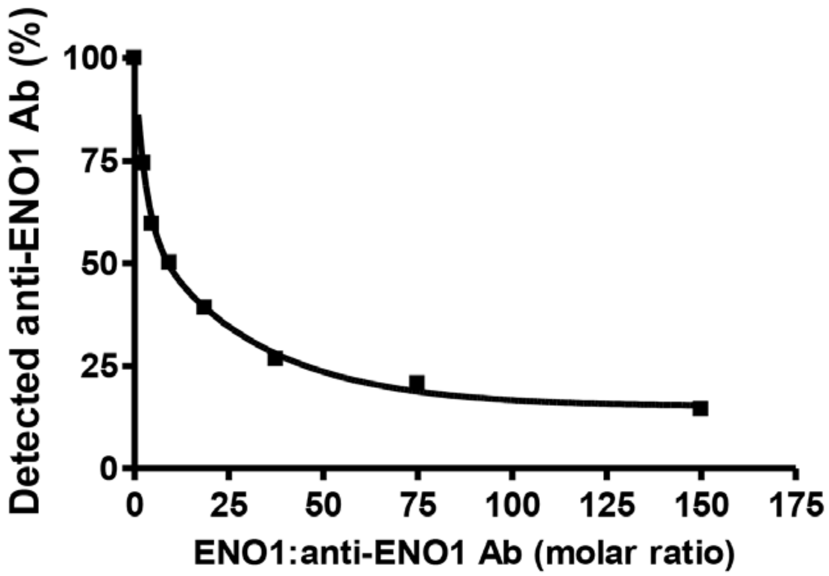

Supplementary Figure S3: Standard curve of ENO1 competitive ELISA. Different concentrations of recombinant ENO1 protein (0.75–48 μg/ml) or PBS were mixed with 1 μg anti-ENO1 Ab as standard concentration and the concentration of detectable anti-ENO1 Ab in the mixtures was determined by ELISA. The detected level of anti-ENO1 Ab in the presence of PBS was set to 100%. The standard curve was established by using the percentage of detected anti-ENO1 Ab at the Y-axis and the molar ratio of added ENO1 protein to anti-ENO1 Ab at the X-axis.

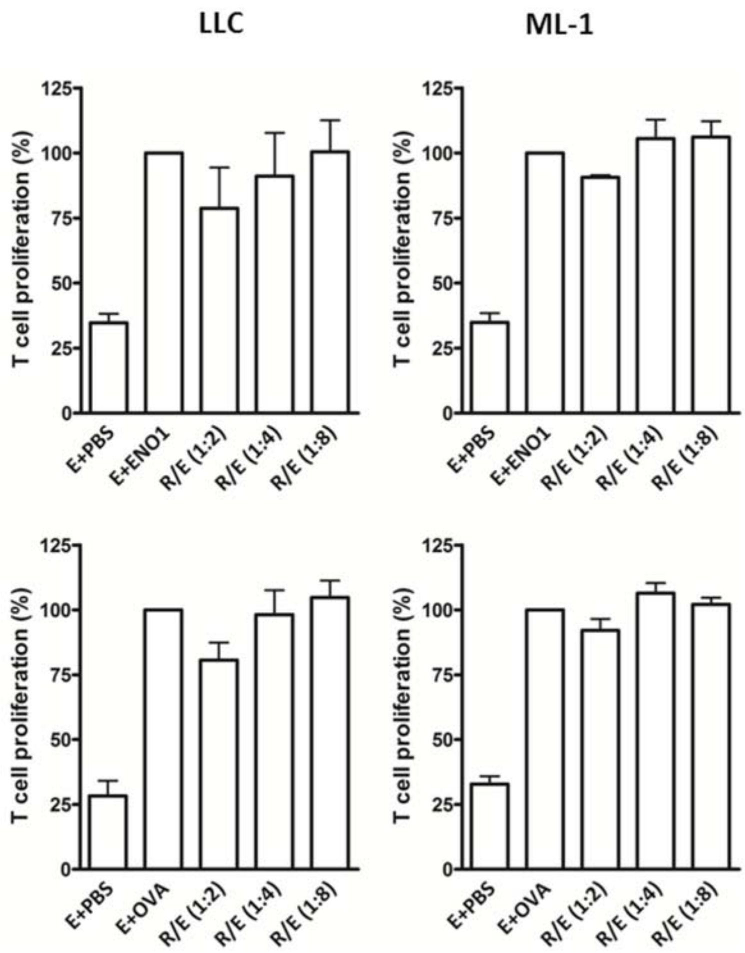

Supplementary Figure S4: The suppressive ability of Treg cells in splenocyte proliferation assay. Mice (n = 5) immunized with ENO1 (top) or OVA (bottom) were challenged with LLC (left) or ML-1 cells (right) as described in Figure 4A. Splenocytes (effector cells) were harvested 28 days after Ag immunization and labeled with CFSE. CFSE-labeled cells were then cultured with ENO1 or OVA as stimulating Ag in the absence or presence of Treg cells, isolated from spleens of tumor-free mice, at 1:2, 1:4, and 1:8 (Treg:effector) ratios. After a 7-day incubation, the cells were harvested and labeled with anti-CD4 Ab. The reduction of CFSE intensity in $CD4^+$ T cells was used to determine the proliferation of effector cells. The proliferation in group of E + Ag was set to 100%. E: splenocytes; ENO1, α-enolase; E + PBS, splenocytes only; R, Treg cells. Bars represent Mean ± SEM.

Supplementary Table S1: Correlation of patients' clinical variables and the level of anti-ENO1 Ab in plasma before surgery

Variable	Total, *n* (%)	>3.3, *n* (%)	≤3.3, *n* (%)	*P*
**Sex**				
Male	38 (44.7)	16 (42.1)	22 (57.9)	0.226
Female	47 (55.3)	26 (55.3)	21 (44.7)	
**Age (y)**				
≤65	56 (65.9)	30 (53.6)	26 (46.4)	0.286
>65	29 (34.1)	12 (41.4)	17 (58.6)	
**Pathology**				
Adeno	72 (85.9)	35 (48.6)	37 (51.4	0.728
Non adeno	13 (14.1)	7 (53.8)	6 (46.2)	
**pTNMStage**				
I/II	65 (76.5)	32 (49.2)	33 (50.8)	0.754
III	20 (23.5)	10 (50.0)	10 (50.0)	
**Tumor Size**				
≤5 cm^3	43 (50.6)	22 (51.2)	21 (48.8)	0.744
>5 cm^3	42 (49.4)	20 (47.6)	22 (52.4)	
**EGFR mutation**				
Yes	46 (61.3)	22 (47.8)	24 (52.2)	0.800
No	29 (38.7)	13 (44.8)	16 (55.2)	
**Q score**				
≤100	37 (59.7)	24 (64.9)	13 (35.1)	0.025
>100	25 (40.3)	9 (36.0)	16 (64.0)	

Adeno, adenocarcinoma; Non adeno, non-adenocarcinoma; y,years.
The median tumor volume was 5 cm^3. The mean Q-score was 100. The median level of anti-ENO1 Ab before surgery was 3.3 µg/ml.

Supplementary Table S2: Multivariate Cox regression analysis to adjust the risk factors for tumor progression

**(A)**	**10% increase of Ab**				
	**Prognostic factor**	**β**	**HR**	**95% CI**	***P***
	**Histological subtype**				
	Non adeno vs Adeno	1.099	3.001	1.247–7.223	0.014
	**pTNM stage**				
	III vs I/II	0.732	2.061	0.886–4.791	0.093
	**Anti-ENO1 level**				
	< 10% vs ≥ 10%	1.005	2.732	1.214–6.149	0.015
	**Tumor size**				
	> 5 cm^3 vs ≤ 5 cm^3	1.113	3.044	1.115–8.309	0.03
**(B)**	**15% increase of Ab**				
	**Prognostic factor**	**β**	**HR**	**95% CI**	***P***
	**Histological subtype**				
	Non adeno vs Adeno	1.01	2.745	1.145–6.583	0.024
	**pTNM stage**				
	III vs I/II	0.596	1.814	0.786–4.188	0.163
	**Anti-ENO1 level**				
	< 15% vs ≥ 15%	0.999	2.716	1.125–6.558	0.026
	**Tumor size**				
	> 5 cm^3 vs ≤ 5 cm^3	1.133	3.105	1.137–8.477	0.027
**(C)**	**20% increase of Ab**				
	**Prognostic factor**	**β**	**HR**	**95% CI**	***P***
	**Histological subtype**				
	Non adeno vs Adeno	0.887	2.429	1.003–5.881	0.049
	**pTNM stage**				
	III vs I/II	0.364	1.439	0.610–3.399	0.406
	**Anti-ENO1 level**				
	< 20% vs ≥ 20%	1.314	3.72	1.241–11.151	0.019
	**Tumor size**				
	> 5 cm^3 vs ≤ 5 cm^3	1.257	3.517	1.272–9.722	0.015

Adeno, adenocarcinoma; Non adeno, non-adenocarcinoma; β, regression coefficient; CI, confidence interval; HR, hazard ratio.

Supplementary Table S3: Multivariate Cox regression analysis to adjust the risk factors for 2-year tumor progression in patients with stage I disease

**(A)**	**10% increase of Ab**				
	**Prognostic factor**	**β**	**HR**	**95% CI**	***P***
	**Histological subtype**				
	Non adeno vs Adeno	1.826	6.211	1.279–30.168	0.024
	**Anti-ENO1 level**				
	$< 10\%$ vs $\geq 10\%$	2.058	7.83	1.359–45.116	0.021
	**Tumor size**				
	> 5 cm^3 vs ≤ 5 cm^3	0.947	2.579	0.526–12.635	0.243
**(B)**	**12% increase of Ab**				
	**Prognostic factor**	**β**	**HR**	**95% CI**	***P***
	**Histological subtype**				
	Non adeno vs Adeno	1.91	6.753	1.378–33.085	0.018
	**Anti-ENO1 level**				
	$< 12\%$ vs $\geq 12\%$	1.921	6.827	1.175–39.655	0.032
	**Tumor size**				
	> 5 cm^3 vs ≤ 5 cm^3	0.863	2.37	0.489–11.497	0.284
**(C)**	**15% increase of Ab**				
	**Prognostic factor**	**β**	**HR**	**95% CI**	***P***
	**Histological subtype**				
	Non adeno vs Adeno	2.265	9.627	1.903–48.701	0.006
	**Anti-ENO1 level**				
	$< 15\%$ vs $\geq 15\%$	2.733	15.386	1.597–148.183	0.018
	**Tumor size**				
	> 5 cm^3 vs ≤ 5 cm^3	0.904	2.47	0.516–11.83	0.258
**(D)**	**20% increase of Ab**				
	**Prognostic factor**	**β**	**HR**	**95% CI**	***P***
	**Histological subtype**				
	Non adeno vs Adeno	2.316	10.13	1.976–51.923	0.005
	**Anti-ENO1 level**				
	$< 20\%$ vs $\geq 20\%$	2.719	15.167	1.534–149.975	0.02
	**Tumor size**				
	> 5 cm^3 vs ≤ 5 cm^3	0.973	2.647	0.548–12.785	0.226

Adeno, adenocarcinoma; Non adeno, non-adenocarcinoma; β, regression coefficient; CI, confidence interval; HR, hazard ratio.

Supplementary Table S4: Multivariate Cox regression analysis to adjust the risk factors for overall survival in patients with stage I disease

**(A)**	**10% increase of Ab**				
	**Prognostic factor**	**β**	**HR**	**95% CI**	***P***
	**Histological subtype**				
	Non adeno vs Adeno	1.472	4.359	0.813–23.368	0.086
	**Anti-ENO1 level**				
	< 10% vs ≥ 10%	1.879	6.548	1.208–35.505	0.029
	**Tumor size**				
	> 5 cm^3 vs ≤ 5 cm^3	0.489	1.631	0.335–7.941	0.544
**(B)**	**12% increase of Ab**				
	**Prognostic factor**	**β**	**HR**	**95% CI**	***P***
	**Histological subtype**				
	Non adeno vs Adeno	1.472	4.359	0.813–23.368	0.086
	**Anti-ENO1 level**				
	< 12% vs ≥ 12%	1.879	6.548	1.208–35.505	0.029
	**Tumor size**				
	> 5 cm^3 vs ≤ 5 cm^3	0.489	1.631	0.335–7.941	0.544
**(C)**	**15% increase of Ab**				
	**Prognostic factor**	**β**	**HR**	**95% CI**	***P***
	**Histological subtype**				
	Non adeno vs Adeno	1.84	6.294	1.096–36.185	0.039
	**Anti-ENO1 level**				
	< 15% vs ≥ 15%	2.429	11.349	1.269–101.47	0.03
	**Tumor size**				
	> 5 cm^3 vs ≤ 5 cm^3	0.331	1.392	.284–6.824	0.684
**(D)**	**20% increase of Ab**				
	**Prognostic factor**	**B**	**HR**	**95% CI**	***P***
	**Histological subtype**				
	Non adeno vs Adeno	1.857	6.404	1.091–37.603	0.04
	**Anti-ENO1 level**				
	< 20% vs ≥ 20%	2.292	9.894	1.124–87.093	0.039
	**Tumor size**				
	> 5 cm^3 vs ≤ 5 cm^3	0.465	1.592	0.324–7.822	0.567

Adeno, adenocarcinoma; Non adeno, non-adenocarcinoma; β, regression coefficient; CI, confidence interval; HR, hazard ratio.

Supplementary Table S5: Patient characteristics

Variable	*n* (%)
**Sex**	
Male	38 (44.7)
Female	47 (55.3)
**Age (y)**	
≤65	56 (65.9)
>65	29 (34.1)
**Pathology**	
Adeno	72 (85.9)
Non adeno	13 (14.1)
**pTNM stage**	
I/II	65 (76.5)
III	20 (23.5)
**Tumor Size**	
≤5 cm^3	43 (50.6)
>5 cm^3	42 (49.4)
**EGFR mutation**	
Yes	46 (61.3)
No	29 (38.7)
**Q score**	
≤100	37 (59.7)
>100	25 (40.3)

Adeno, adenocarcinoma; Non adeno, non-adenocarcinoma; y,years.
The median tumor volume was 5 cm^3. The mean Q-score was 100.
